# Mapping private pharmacies and their characteristics in Ujjain district, Central India

**DOI:** 10.1186/1472-6963-11-351

**Published:** 2011-12-28

**Authors:** Yogesh D Sabde, Vishal Diwan, Vivek S Saraf, Vijay K Mahadik, Vinod K Diwan, Ayesha De Costa

**Affiliations:** 1 R.D.Gardi Medical College, Ujjain, 456006, India; 2 Division of Global Health (IHCAR) Karolinska Institutet, Stockholm, 17177, Sweden; 3 National Center for Human Settlement and Environment, Bhopal, 462016, India

## Abstract

**Background:**

In India, private pharmacies are ubiquitous yet critical establishments that facilitate community access to medicines. These are often the first points of treatment seeking in parts of India and other low income settings around the world. The characteristics of these pharmacies including their location, drug availability, human resources and infrastructure have not been studied before. Given the ubiquity and popularity of private pharmacies in India, such information would be useful to harness the potential of these pharmacies to deliver desirable public health outcomes, to facilitate regulation and to involve in initiatives pertaining to rational drug use. This study was a cross sectional survey that mapped private pharmacies in one district on a geographic information system and described relevant characteristics of these units.

**Methods:**

This study of pharmacies was a part of larger cross sectional survey carried out to map all the health care providers in Ujjain district (population 1.9 million), Central India, on a geographic information system. Their location vis-à-vis formal providers of health services were studied. Other characteristics like human resources, infrastructure, clients and availability of tracer drugs were also surveyed.

**Results:**

A total 475 private pharmacies were identified in the district. Three-quarter were in urban areas, where they were concentrated around physician practices. In rural areas, pharmacies were located along the main roads. A majority of pharmacies simultaneously retailed medicines from multiple systems of medicine. Tracer parenteral antibiotics and injectable steroids were available in 83.7% and 88.7% pharmacies respectively. The proportion of clients without prescription was 39.04%. Only 11.58% of staff had formal pharmacist qualifications. Power outages were a significant challenge.

**Conclusion:**

This is the first mapping of pharmacies & their characteristics in India. It provides evidence of the urban dominance and close relationship between healthcare provider location and pharmacy location. The implications of this relationship are discussed. The study reports a lack of qualified staff in the presence of a high proportion of clients attending without a prescription. The study highlights the need for the better implementation of regulation. Besides facilitating regulation & partnerships, the data also provides a sampling frame for future interventional studies on these pharmacies.

## Background

Private pharmacies in low and middle income countries are one of the first points of access for communities to healthcare advice and medication. Studies have reported that it is routine for people to seek the advice of pharmacists and medical shop attendants for common ailments [1,2]. Numerous studies have established the important role of pharmacists in providing medications and advice for diarrhea [3], respiratory tract infections [4,5], tuberculosis [6,7], asthma [8], and sexually transmitted diseases [9,10] in populations around the world. Such consultations are convenient and save time, money and the opportunity cost of waiting to see a physician [1,2].

India has one of the most highly privatized health care systems in the world in terms of finance and delivery [11]. The private health sector in the country is complex; providers vary from being highly qualified specialists to unqualified persons, practicing different systems of medicine in diverse organizational setups. There has been no published estimate of pharmacies operating within the country's private sector. However it is reasonable to suggest that a large proportion of pharmacies are in the private sector, given that 72% of the country's health care expenditure is in the private sector [12]. Also a recent study of a province of central India indicated that three quarters of all qualified paramedical staff (including pharmacists) were in the private sector [13].

There have been a number of reports from India studying the dispensing patterns at pharmacies [14], antibiotic prescribing at these outlets [15] and treatment of different conditions by drug sellers [8,16,17]. There have been few studies that list, map and study the characteristics of private pharmacies reported in the literature. This study aims to survey all private pharmacies in one district of Central India, and map them on a geographical information system (GIS) to study their location including their rural-urban distribution, as well as their relationship to major roadways and the location of other healthcare providers. In addition, characteristics of these private pharmacies in terms of human resources, clients, availability of tracer drugs and infrastructure were studied.

## Methods

### Setting

**Figure 1:**
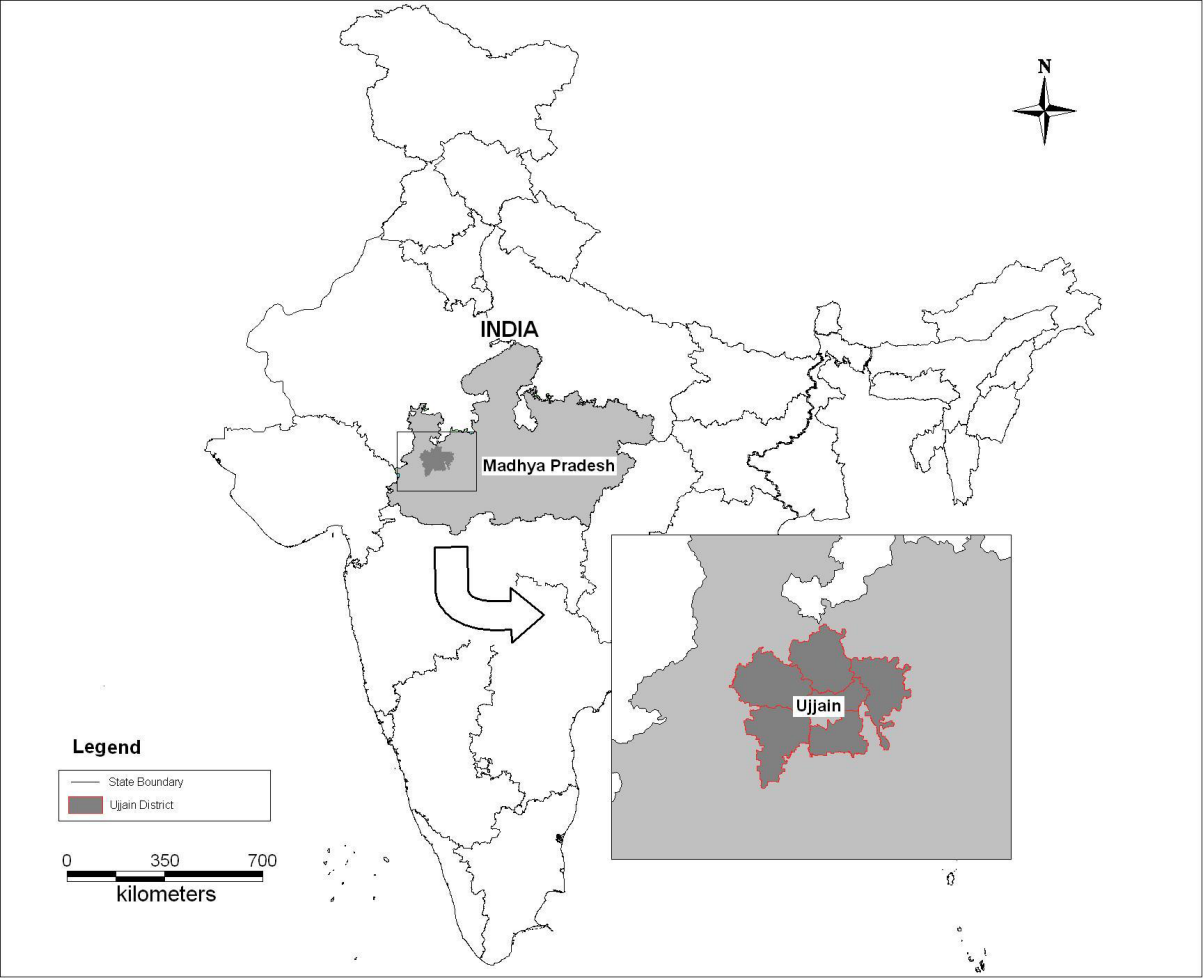
**Location of Ujjain in India**.

This study was conducted in Ujjain district, Madhya Pradesh, Central India. Madhya Pradesh (MP), which is one of India's largest provinces, is divided into 50 administrative districts each with a population of a little over a million. Ujjain, spread over 6091 sq km, on the western flank of the province, is one such district (Figure 1). It has a population of 1.9 million, 61% of which is rural. A quarter of the population belongs to schedule castes [18]. Scheduled castes (and scheduled tribes) are those communities that were historically subject to social disadvantage and exclusion. They are accorded special status by the Constitution of India (they are listed in a schedule) and are recipients of special social benefits as part of a national program of positive affirmation [19]. The district literacy rate is 72% and infant mortality rate is 51.9/1000 live births, close to the national average [18]. Agriculture is the mainstay of the district economy. Ujjain city (0.5 million inhabitants) is the largest town and is the administrative headquarters of the district.

### Survey of private pharmacies

This survey was a part of larger project to study and map all the health care providers in Ujjain district. As there are no existing comprehensive records of pharmacies in the province, a primary survey was necessary.

In this study, the term 'Pharmacy' included every store or shop where drugs are dispensed, bearing a sign with words "Pharmacy", "Pharmacist," "Dispensing Chemist" or "Pharmaceutical Chemist" or the term 'dawainya' (medicines) in Hindi [20,21]. Private pharmacies refer to privately owned pharmacies that sell the drugs for profit, paid for out-of-pocket by clients. Private pharmacies in this study were classified as being 'stand alone' if they were independent pharmacies and as 'attached' if they were part of a larger institution, usually a hospital. Public pharmacies, which were excluded in the study, were state owned and attached to health institutions in the tiered public health system.

The field work was done by surveyors (total 10) who had a master's degree in social work. These surveyors were employed by the medical school for ongoing research work in the local community. The surveyors were trained by the research team at the medical school to identify private pharmacies in the district, meet persons in the pharmacy and administer a short questionnaire to them (see Additional files 1 and 2). The survey instrument was a structured questionnaire that included information on the exact location of the private pharmacy, clients, medical systems for which drugs were dispensed, the availability of certain tracer drugs, infrastructure and human resources. Five tracer medicines were selected to study availability of commonly prescribed drugs viz. cotrimoxazole, amoxicillin+clavulanate, anti-TB drugs (rifampicin/INH), inj. cefotaxime and inj. dexamethasone. Data collected from a provider survey earlier showed that these were commonly prescribed drugs in the study area; hence their selection as tracer drugs.

The basic unit of the survey was a village in rural areas and a ward in urban areas. (A ward is the smallest administrative division in a town with a population of 20,000-50,000). Rural and urban areas were defined as per classification in the Census of India [22]. Some fragmented initial information on pharmacies was available through the local district pharmacy associations, which served as a starting point for the survey. Surveyors first contacted the public health worker, cre`che worker, panchayat (village self-government), schoolteacher, or an influential person, in the village to enquire about the presence of a pharmacy in the rural areas. In urban areas, pharmacy boards are prominently displayed, surveyors in addition checked for the presence of other units in the ward from other pharmacists, besides referring to the list from the pharmacy association. If a pharmacy was closed or the person in the pharmacy was not available at the time of visit, then surveyors visited again during the working hours as mentioned on the display board. Less than 2% of such pharmacies could not be studied as they were closed after a second visit. The process was completed between June and Oct 2009.

### Mapping of pharmacies onto a GIS

All private pharmacies detected in the study district were plotted onto a digitized map of Ujjain. To demonstrate the geographical distribution of pharmacies, the following two maps were used: (i) geo-referenced digitized map of Ujjain district developed by Deshpande *et al*. in 2004 [23] (ii) a detailed geo-referenced digitized base map of Ujjain city. The first was used to depict the location of pharmacies in rural and urban areas (excluding Ujjain city area). The second map - that was developed as a part of this study, was used to visualize pharmacy and health provider locations within the city.

Ujjain city base maps were procured from the office of the Town and Country Planning Directorate and the Ujjain Municipal Corporation. These maps were first scanned using calibrated large-size optical scanners and then onscreen digitization was done using AutoCad Map software to extract relevant features such as city boundary, ward boundary, roads, and important land marks. The Survey of India topographical sheets on the scale of 1:50,000 were used for geo-referencing. The cross verification of geo-referencing on ground was subsequently performed using hand held global positioning system (GPS) at random locations for precision. Source maps details were updated using the Google maps and CartoSAT2 satellite data. The latter is a high resolution remote sensing image availed from Indian Space Research Organization (ISRO) with some verification from Ujjain Municipal Corporation officials.

At the time of the data collection of the pharmacies, the field surveyors were provided this updated map of the city to mark the respective pharmacies by referring to the surroundings and adjoining details shown on the map. This method of marking on a geo-referenced base map helped ascertain accuracy of locations. This was further fine-tuned with the support of geopositioning. Geo-position of pharmacies as a separate layer on base map was done using AutoCAD Map GIS software. A database of pharmacies was prepared using the database management software - MS Access. The maps and data base were imported into Map info software. The data tables included unique ID codes for each pharmacy, which were used to relate pharmacy location on the map with the relevant data record from the database.

The relationship between pharmacies and providers was studied using concentric ring buffers around provider locations. In our initial mapping we visualized that most pharmacies were located within a 250 m radius from the provider clinics. Therefore a distance of 50 m was deemed appropriate for concentric ring buffers around the providers. Thus five concentric ring buffers of were plotted at a distance of 50, 100, 150, 200 and 250 meters respectively.

### Data management and Analysis

The data were compiled ward wise in urban areas and village wise in rural areas. Appropriate codes were given according to each ward/village in all the blocks. The data were entered in to MS Access spreadsheets and transferred in to PASW Statistics 18.0. Basic descriptive statistics were presented. Tests of significance for proportion were run. The distribution of pharmacies as visualized in the GIS developed is presented.

### Ethical approval

The study was approved by the Ethics Committee of R.D. Gardi Medical College, Ujjain.

## Results

A total of 475 private pharmacies were identified in the district, with more than three-quarter (77.7%) in urban areas. The overall density of pharmacies was 28 per 100,000 population (urban/rural distribution of 58.39 and 8.40 per 100 000 respectively). Most pharmacies (95%) were independent 'stand alone' units.

### Distribution of pharmacies

**Figure 2:**
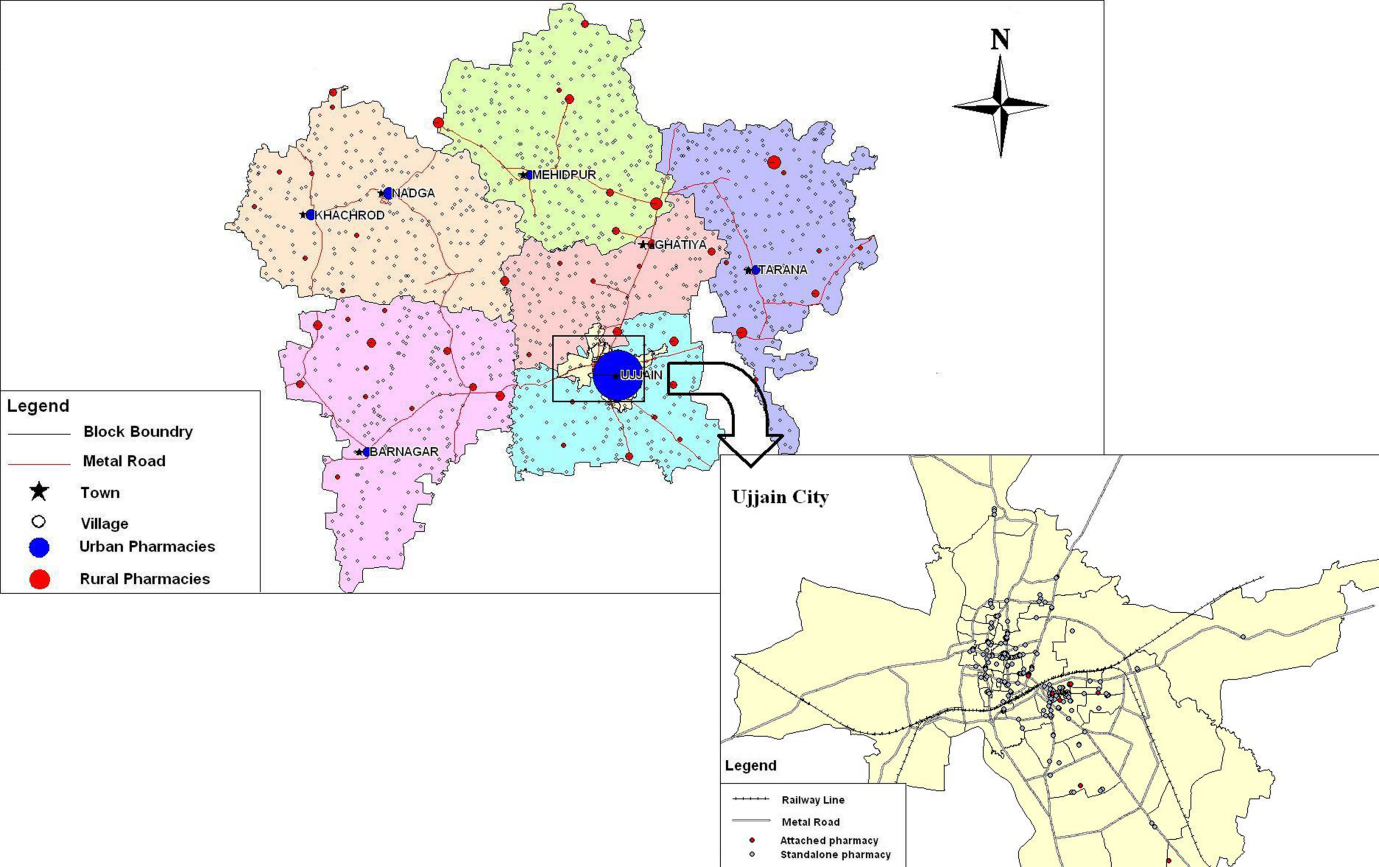
**Distribution of pharmacies in Ujjain district**.

**Figure 3:**
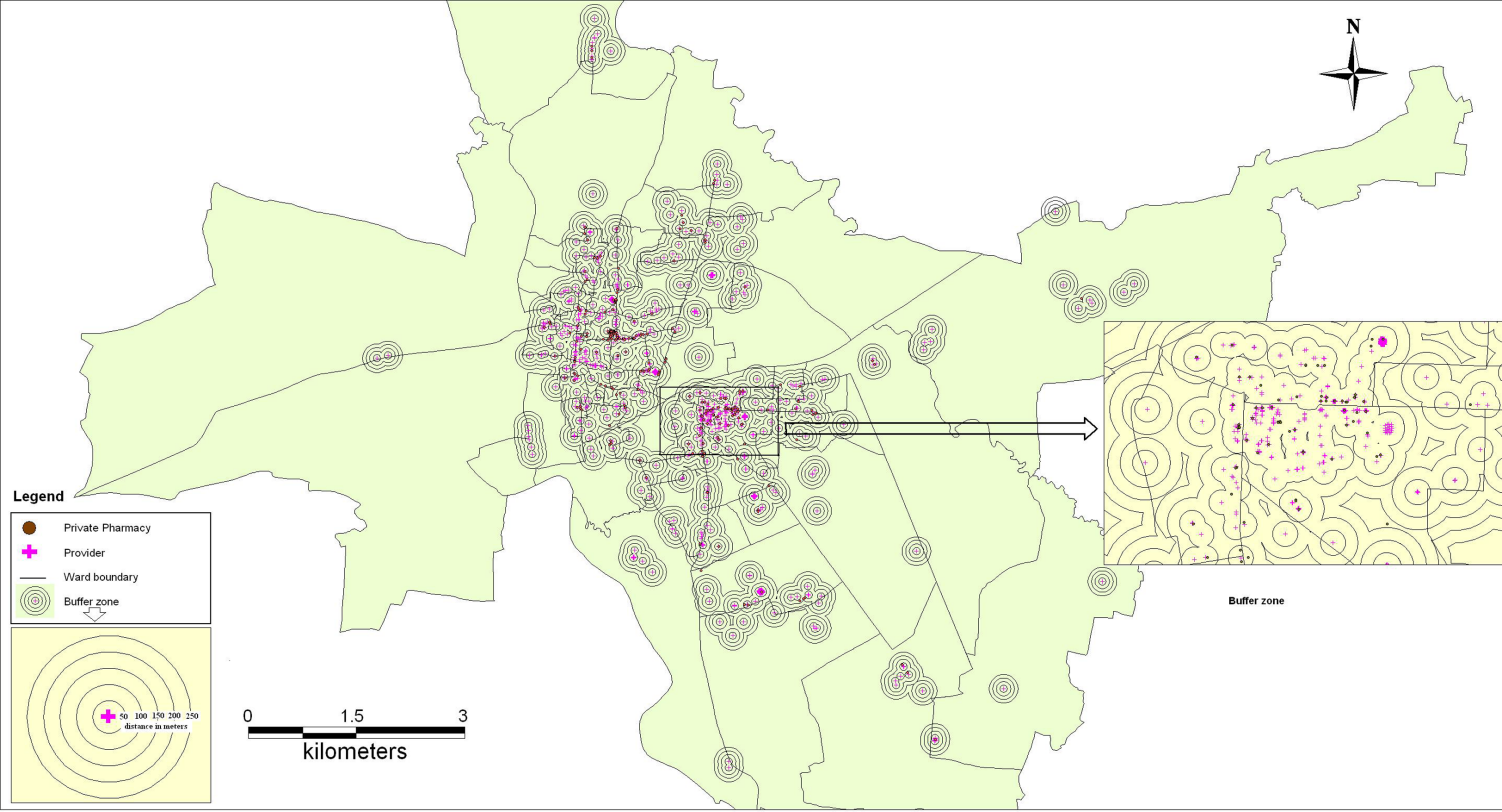
**Relationship between pharmacies and health care providers**.

The distribution of the pharmacies is as shown in table 1. The distribution of private pharmacies mirrors the distribution of qualified physicians in the district, 23% rural and 77% urban (unreported data from the same survey). The spatial location of pharmacies in Ujjain district is shown in Figure 2 which shows a concentration in the urban areas. In rural areas, pharmacies were located on main roads. Figure 3 shows the relationship between the location of healthcare providers and the location of private pharmacies in Ujjain city. Three quarters (78%) of all pharmacies were within 0-50 m radius from a health care provider (out patient clinic or hospital) while a further 17% were located between 51-100 m from a provider.

**Table 1:** Distribution of private pharmacies in Ujjain district.

Location	Rural n (%)	Urban n (%)
Total private pharmacies	88 (22.3)	387* (77.7)
Stand alone pharmacies	82 (93.2)	370 (95.6)
Attached	6 (6.8)	17 (4.4)

*300 in Ujjain city

### Clients

Table 2 shows the reported average number of clients utilizing pharmacies in urban and rural areas per day. The proportions of clients without prescriptions (Table 2) averaged 40% in stand alone pharmacies

**Table 2:** Clients in the urban and rural pharmacies

Location of pharmacy	Urban	Rural	Total
**Average no. of clients per day**	(n = 372)	(n = 85)	(n = 457)
Total	98.61	46.84	88.98
Stand alone (n = 435)	98.40	43.51	88.43
Attached (n = 22)	103.25	90.67	99.82
**Proportion of clients with doctor's prescription**	(n = 311)	(n = 85)	(n = 396)
Total	62.04	56.99	60.96
Stand alone(n = 374)	60.67	56.62	59.84
Attached(n = 22)	86.85	61.80	80.10

### Medicines dispensed

A majority of the pharmacies 462 (97.3%) principally dispensed drugs used in modern (allopathic system) medicine. Medicines used in other systems of medicine (Ayurveda, Homeopathy and Unani) were simultaneously dispensed in 288 (60.6%) pharmacies. The proportion of such pharmacies was significantly higher (72.7%) in rural areas than urban areas (57.9%). Drugs for veterinary use were dispensed in all rural pharmacies and in 8.5% of urban pharmacies. The availability of different tracer drugs is shown in table 3. Vaccines listed in national immunization schedule (TB, oral polio, DPT and measles) were available in only 8.5% of urban and 5.7% of rural pharmacies respectively.

**Table 3:** Availability of tracer drugs in urban and rural pharmacies.

Tracer drugs	Urban n = 358		Rural n = 83		Total n = 441	
	No	%	No	%	No	%
Cotrimoxazole	323	(90.2)	75	(90.4)	398	(90.2)
Amoxcillin + Clavulanate	303	(84.6)	68	(81.9)	371	(84.1)
Anti TB -Rifampicin, INH	235	(65.6)	30	(36.1)	265	(60.1)
Inj. Cefotaxime	298	(83.2)	71	(85.5)	369	(83.7)
Inj. Dexamethasone	315	(88.0)	76	(91.6)	391	(88.7)

### Human resources in the pharmacies

On enquiring about human resources, 64 (14%) pharmacies refused to provide details of their staffing. The remaining 393 pharmacies had 679 staff, 12 (1.8%) of whom were women. Only 81 (11.58%) had the minimal formal qualification in pharmacy mandated by law to practice as a registered pharmacist [21]. Most of these qualified persons (88%) worked in urban pharmacies

### Infrastructure

Refrigerators were available in 284 (73.39%) urban and 18 (20.40%) rural pharmacies. Two thirds of these reported experiencing power outages for a minimum of four hours everyday, yet only 77 had a back up source to power the refrigerator when necessary.

## Discussion

This is the first reported survey of characteristics and distribution of private pharmacies in an Indian district. The distribution of private pharmacies reflects the distribution pattern of private physicians and hospitals in Madhya Pradesh province [13]. This earlier study had reported ten times as many physicians and twenty times as many beds per unit population in urban than in rural areas. With pharmacies in Ujjain district, the urban rural distribution pattern was similar, with 75% of mapped pharmacies in urban areas. The availability per unit population was six times more in urban areas.

Figure 3 indicates the location of pharmacies in proximity to physician practices/hospitals in urban areas. Besides difficult access to these pharmacies for rural populations, there are other implications of such a skewed urban centric distribution [24]. Too many private pharmacies clustered around doctors' clinics/hospitals, implies fierce competition among them [2]. This could encourage potential malpractice (including pay backs to prescribers), compromises in human resources and in quality of medicines, particularly in a weakly regulated environment [24]. On the other hand, it could also imply convenient access to a wide range of medicines for urban clients (a third of the district) consulting at physician practices. Regulation of the minimal distance between pharmacies in some provinces has been attempted, but has not been successful [24].

The concentration of pharmacies around physician practices particularly in urban areas could also indicate that pharmacies in these areas are not points of first contact for patients, but rather that physicians are, and that pharmacies choose locations around these practices.

The GIS indicates the location of pharmacies on major roads that course through the district and the city. Concern has been raised about how this could affect the quality of medicines stored in pharmacies, as most do not have any air conditioning, and were open to the road throughout the working day, allowing heat, dust and dirt into the premises [24].

Urban and rural pharmacies in the district had a median number of 70 and 40 clients a day, respectively. There was no major difference in client numbers between 'stand alone' and 'attached' pharmacies in urban areas. In rural areas, attached pharmacies seemed to be better patronized than the stand alone pharmacies. In this study, nearly half of all clients purchased medicines without a prescription. A recent study from Tamil Nadu reported two thirds of clients purchased drugs from pharmacies without prescription in two study districts [14]. In attached pharmacies, where prescriptions from the parent institution were most likely to be received, the proportion of non- prescription clients was low both in rural and urban areas.

The availability of tracer medicines did not differ significantly between urban and rural pharmacies. The oral antibiotic combination amoxicillin + clavulanic acid which is used in special situations was available in 84.1% of all pharmacies in the district. Third generation injectable cephalosporin i.e. cefotaxime was also available similarly i.e 83.7%. Availability of injectable steroid dexamethasone was as high as 88.7% in the district. Though these are prescription drugs, their availability indicates that they could easily be dispensed over the counter. A recent report from south India indicates similar findings in pharmacies there. The study reports that 42% of antibiotics dispensed to clients were given based on the drug sellers recommendation, 37% were dispensed on client request while only 21% were dispensed in response to a prescription [14]. A much earlier study reported only 2.9% of antibiotics were given based on the pharmacists recommendation while 21% were client requested, [25] much lower figures than seen in the more recent study. There have been many such reports from other neighboring countries as well [26]. Another important observation in this study was that only 12% of staff in the pharmacies were trained pharmacists. This situation with regard to human resources was worse than reports from other parts of the country. In south India, 50% of pharmacies were reported to have trained pharmacists [27]. The regulations governing pharmacy practice in India state that, any prescribed drug should be supplied, only by or under the personal supervision of a registered pharmacist. The minimum qualification for registration as a pharmacist is a "Diploma in Pharmacy" (D.Pharm) or "Degree in Pharmacy" (B.Pharm) from an institution approved by the Pharmacy Council of India [20,21]. While theoretically this means that every pharmacy must have a trained pharmacist (as a legal requirement), in practice few pharmacists were on location, and dispensing was often done by a supporting person who is less qualified [28]. Similar findings of inadequately qualified staff have been reported from other studies in India [2], as well as from other low income countries [29,30]. In a study in New Delhi India poor education of the dispensing pharmacists was identified as an important contributor for irrational use of antibiotics [31]. Another study from Nepal also reported that the higher educated pharmacists had a more correct knowledge about contraceptive products [32]. Similar reports of poor practices by pharmacy staff have been documented in different studies from the country [6,8,14]. Having unqualified persons dispensing medications in pharmacies in settings where so much of treatment is client requested only furthers already high levels of irrational drug use. This issue thus requires attention particularly in the light of recent reports of high level resistance in India [33]. Apart from the regulations [20,21] educational programmes for the qualified pharmacists have also been suggested by the other reports [31,34]. There are other factors which influence pharmacy behavior including client expectation, economic incentives, local physician behavior and most importantly, the level of effective regulation [1].

Community pharmacy is still evolving in India, and the role of community pharmacists has not been defined beyond dispensing. Pharmacists have not been mentioned in the National Health Policy [35] or the National Pharmaceutical Policy [36], possibly because of a lack of clarity on their role beyond supply of pharmaceutical products [24]. The current regulatory framework does not recognize the need for clinical pharmacists at the national level [37]. Public awareness on the importance of a pharmacist is absent - they are viewed as traders and not as professionals [24,38]. There has been some critique of pharmacy education in India as preparing pharmacists to take on roles in industry and not in community pharmacy [38,39]. Trained pharmacists prefer the former as it is more remunerative, also community pharmacy is not yet considered a profession in its own right.

### Methodological considerations

While we have used the word 'pharmacy' for outlets that sell medicines, these are legally categorized as 'Chemist & Druggists' or 'Pharmacies'. Both kinds of outlets are required to employ the services of a qualified pharmacist. However Chemist & Druggists' do not prepare (compound) medications against prescriptions while the pharmacies are allowed to do so [20,21,28]. Both are required to display appropriate signage, which was used in this study to identify them. Chemist & Druggists were the majority in this study (99%). There exist in addition, general stores that sell a few medicines like analgesics, antipyretics, lozenges etc; - these have been excluded from the purview of this study.

With regard to human resources, we sought details of the person/s in the pharmacy at the time of interview i.e. the person who interacted with clients over the counter that day. There could be other persons on other days whose details are not reflected in the results. Similarly information provided to the surveyors was dependent on the person available at the pharmacy on the day of survey

The number of clients, with and without prescriptions were self-reported by respondents and not observed by the researchers.

Public pharmacies were not enumerated or mapped in this study. Not more than 50 such would be expected in Ujjain district.

## Conclusions

This study surveyed and mapped all private pharmacies in the central Indian district of Ujjain. It provides information on the location of private pharmacies, and evidence of close relationships between provider locations and pharmacy locations. The study reports of human resources and other characteristics were in line with other previously published literature and provide evidence for the need for the better implementation of regulation of pharmacies and the importance of supporting the evolution of regulated community pharmacy in the country.

## Competing interests

The authors declare that they have no competing interests.

## Authors' contributions

YS, carried out the fieldwork, analysed the results and drafted the manuscript. VD initiated the concept, participated in the design and coordination of the study and in the analysis and interpretation of results and participated in drafting and finalising the manuscript. VS was responsible for the development of GIS map and customized software. VM and VKD participated in the development of design in the initial stages of the study and reviewed the manuscript. AD participated in developing the concepts, participated in the design of the study, analysed the results and helped in drafting the manuscript. All authors have read and approved the final manuscript.

## Pre-publication history

The pre-publication history for this paper can be accessed here:

http://www.biomedcentral.com/1472-6963/11/351/prepub

## Supplementary Material

Additional file 1 **Questionnaire 1**. Field questionnaire for pharmacy infrastructure. Click here for file

Additional file 2 **Questionnaire 2**. Field questionnaire for tracer medicines available in pharmacy. Click here for file

## References

[B1] Goel P Ross-Degnan D Berman P Soumerai S Retail pharmacies in developing countries: a behavior and intervention framework Soc Sci Med 1996 42 1155 61 10.1016/0277-9536(95)00388-6 8737433

[B2] Kamat VR Nichter M Pharmacies, self-medication and pharmaceutical marketing in Bombay, India Soc Sci Med 1998 47 779 94 10.1016/S0277-9536(98)00134-8 9690824

[B3] Benjamin Sokar-Todd H Smith F Management of diarrhoea in community pharmacies in Alexandria, Egypt J Soc Adm Pharm 2003 20 32 8

[B4] Chalker J Chuc NT Falkenberg T Tomson G Private pharmacies in Hanoi, Vietnam: a randomized trial of a 2-year multi-component intervention on knowledge and stated practice regarding ARI, STD and antibiotic/steroid requests Trop Med Int Health 2002 7 803 10 10.1046/j.1365-3156.2002.00934.x 12225513

[B5] Tumwikirize WA Ekwaru PJ Mohammed K Ogwal-Okeng JW Aupont O Management of acute respiratory infections in drug shops and private pharmacies in Uganda: a study of counter attendants' knowledge and reported behaviour East Afr Med J 2004 Suppl S33 40 15125114

[B6] Rajeswari R Balasubramanian R Bose MS Sekar L Rahman F Private pharmacies in tuberculosis control--a neglected link Int J Tuberc Lung Dis 2002 6 171 3 11931419

[B7] Lambert ML Delgado R Michaux G Volz A Van Der Stuyft P Tuberculosis control and the private health sector in Bolivia: a survey of pharmacies Int J Tuberc Lung Dis 2004 8 1325 9 15581200

[B8] Van Sickle D Management of asthma at private pharmacies in India Int J Tuberc Lung Dis 2006 10 1386 92 17167957

[B9] Leiva A Shaw M Paine K Manneh K Mcadam K Mayaud P Management of sexually transmitted diseases in urban pharmacies in The Gambia Int J STD AIDS 2001 12 444 52 10.1258/0956462011923471 11394980

[B10] Ramos MC Da Silva RD Gobbato RO Da Rocha FC De Lucca Junior G Vissoky J Cestari T Filgueiras A Pharmacy clerks' prescribing practices for STD patients in Porto Alegre, Brazil: missed opportunities for improving STD control Int J STD AIDS 2004 15 333 6 10.1258/095646204323012832 15117504

[B11] Radwan I (2005) India- Private Health Services for the Poor[online]. http://siteresources.worldbank.org/HEALTHNUTRITIONANDPOPULATION/Resources/281627-1095698140167/RadwanIndiaPrivateHealthFinal.pdf (Accessed 25 April 2011)

[B12] Ministry of Health and Family Welfare & Government of India (2005) National Health Accouts for India 2001-02 [online]. http://www.who.int/nha/NHA_India_NHA_2001-02.pdf (Accessed 09 December 2012)

[B13] De Costa A Diwan V 'Where is the public health sector?' Public and private sector healthcare provision in Madhya Pradesh, India Health Policy 2007 84 269 76 10.1016/j.healthpol.2007.04.004 17540472

[B14] Basak SC Sathyanarayana D Evaluating medicines dispensing patterns at private community pharmacies in Tamil Nadu, India Southern Med Review 2010 3 27 31

[B15] Kotwani A Holloway K Trends in antibiotic use among outpatients in New Delhi, India BMC Infect Dis 2011 11 1 99 10.1186/1471-2334-11-99 21507212 PMC3097160

[B16] Pathak D Pathak A Marrone G Diwan V Lundborg CS Adherence to treatment guidelines for acute diarrhoea in children up to 12 years in Ujjain, India--a cross-sectional prescription analysis BMC Infect Di 2011 11 32 10.1186/1471-2334-11-32 PMC3045317 21276243

[B17] Pandey A Health care options in childhood ARI before hospital care Indian J Public Health 2002 46 51 56 12653002

[B18] Registrar General and Census Commissioner & Government of India (2011) Census of India

[B19] Constitution of India (As modified up to the 1^st ^December, 2007) Government of India Ministry of Law and Justice. New Delhi http://lawmin.nic.in/coi/coiason29july08.pdf Accessed on 29.10.11

[B20] Govt of India (1945) (ammended June 2005). The Drugs and Cosmetics Act http://cdsco.nic.in/html/copy%20of%201.%20d&cact121.pdf accessed on 01.10.11

[B21] Govt of India (1948) The Pharmacy Act. Chapter 4. section 31. Qualifications for entry on first register http://www.pci.nic.in/RulesRegulations/PharmacyAct1948/Chapter4.aspx Accessed on 01.10.11

[B22] Commissioner (2011), R.G.A.C Census of India, census terms[online]. http://censusindia.gov.in/Data_Products/Library/Indian_perceptive_link/Census_Terms_link/censusterms.html (Accessed 25 April 2011)

[B23] Deshpande K Ravi Shankar Diwan V Lonnroth K Mahadik VK Chandorkar RK Spatial pattern of private health care provision of Ujjain, India: a provider survey processed and analysed with a Geographical Information System Am J Public Health 2004 68 211 22 10.1016/j.healthpol.2003.09.012 15063020

[B24] Who India Country Office (2007) A report on Challenges & Opportunities for Pharmacists in Health Care in India http://www.whoindia.org/LinkFiles/Essential_Drugs_and_Medicines_Report_on_CO_for_Pharmacists_in_HC_in_India.pdf Accessed on 25.09.1122224019

[B25] Dua V Kunin CM White LV The use of antimicrobial drugs in Nagpur, India. A window on medical care in a developing country Soc Sci Med 1994 38 717 24 10.1016/0277-9536(94)90462-6 8171350

[B26] Smith F The quality of private pharmacy services in low and middle income countries: a systematic review Pharm World Sci 2009 31 351 61 10.1007/s11096-009-9294-z 19343530

[B27] Basak SC Prasad G Arunkumar A Senthilkumar S An attempt to develop community pharmacy practice: results of two surveys and two workshops conducted in Tamil Nadu Ind J Pharm Sci 2005 67 362 367

[B28] Basak SC Arunkumar A K M Community Pharmacists attitudes towards use of rural medicine in India- An analysis of the current situation Int Pharm J 2002 16 32 35

[B29] Wolf-Gould CS Taylor N Horwitz SM Barry M Misinformation about medications: in rural Ghana Soc Sci Med 1991 33 83 9 10.1016/0277-9536(91)90459-P 1882245

[B30] Stenson B Syhakhang L Eriksson B Tomson G Real world pharmacy: assessing the quality of private pharmacy practice in the Lao People's Democratic Republic Soc Sci Med 2001 52 393 404 10.1016/S0277-9536(00)00142-8 11330774

[B31] Kotwani A Wattal C Joshi PC Holloway K Irrational use of antibiotics and role of the pharmacist: an insight from a qualitative study in New Delhi, India J Clin Pharm Therpublished online: 23 Aug 2011 http://onlinelibrary.wiley.com/doi/10.1111/j.1365-2710.2011.01293.x/abstract Accessed on 01.10.11 10.1111/j.1365-2710.2011.01293.x 21883328

[B32] Shrestha A Kane TT Hamal H Contraceptive social marketing in Nepal: consumer and retailer knowledge, needs and experience J Biosoc Sci 1990 22 3 305 22 2401674 10.1017/s002193200001868x

[B33] Walsh TR Weeks J Livermore DM Toleman MA Dissemination of NDM-1 positive bacteria in the New Delhi environment and its implications for human health: an environmental point prevalence study Lancet Infect Dis 2011 11 5 355 62 10.1016/S1473-3099(11)70059-7 21478057

[B34] Goel PK Ross-Degnan D Mclaughlin TJ Soumerai SB Influence of location and staff knowledge on quality of retail pharmacy prescribing for childhood diarrhea in Kenya Int J Qual Health Care 1996 8 519 26 9007601 10.1093/intqhc/8.6.519

[B35] Ministry of Health and Family Welfare (2002) The National Health Policy 2002 (India). Government of India

[B36] Department of Chemicals and Petrochemicals (2006). National Pharmaceuticals Policy Government of India

[B37] Mangasuli S Rajan S Khan SA A decade of pharmacy practice education in India Am J Pharm Educ 2008 72 16 10.5688/aj720116 18322578 PMC2254242

[B38] Lal LS Rao PG Clinical pharmacy education in India Am J Health Syst Pharm 2005 62 1510 1 10.2146/ajhp040482 15998934

[B39] Basak SC Sathyanarayana D Pharmacy education in India Am J Pharm Educ 2010 74 68 10.5688/aj740468 20585429 PMC2879119

