# Supplementary material for: Mapping private pharmacies and their characteristics in Ujjain district, Central India
Source: BMC Health Serv Res. 2011 Dec 28;11:351. doi: 10.1186/1472-6963-11-351 (PMC3272060; doi:10.1186/1472-6963-11-351)
Supplement: Additional file 1 — Questionnaire 1. Field questionnaire for pharmacy infrastructure. [file 1472-6963-11-351-S1.PDF]

R.D. GARDI MEDICAL COLLEGE, UJJAIN

FORM "P"

Survey of Pharmacies, Medical Shops and Drug Distributors of Ujjain District

उज्जैन जिलें की दवा दुकानों का सर्वेक्षण

Sr. No. / क्रमांक ______ Code No. / कोड नं. ______ Block / ब्लॉक ____________ Village / गांव ____________ Ward / वार्ड ____________ City / शहर ____________

Name and Complete address of Pharmacy/ Medical Shop :
दवा दुकान का नाम व पूरा पता

Phone No. (Landline)
फोन नं

Open 24 Hrs. : Yes/ No क्या दुकान 24 घंटे खुली रहती है हाँ / नहीं

If No : यदि नहीं तो दुकान
Opening Hrs on working days / खुलने का समय ....
Closing Hrs. on working days /बंद होने का समय ....
Lunch break if any/ भोजन अवकाश यदि हो तो
from / कब से .... to / कब तक

Non working day in a week : सप्ताह मे दुकान बंद रहने का दिन ....

No. of approximate customers per day ....
दुकान में प्रतिदिन ग्राहकों की औसत संख्या

a) How many of them have doctor's prescription ? ....
इनमें से कितनों के पास डॉक्टर की पर्ची / चिट्ठी होती है ?

b) How many ask medicine by the name of medicine ?
कितने मरीज दवाई का नाम लेकर दवाई मांगते है ? ....

c) How many ask medicine by symptoms/ Complaints ?
कितने मरीज रोग के लक्षण बताकर उस रोग की दवा मांगते है ? ....

Is there any refrigerator/freezer in this pharmacy Yes/No
फीज उपलब्ध हाँ / नहीं

Average duration of power failure (load shedding) .... Hrs.
बिजली जाने का औसत समय .... घंटा

Generator/ Invertor backup for refrigerator/ freezer :
Available / Not available
फींज के लिए जनरेटर सुविधा उपलब्ध हाँ / नहीं

Computer Available / Not available
कम्प्युटर उपलब्ध है हाँ / नहीं

Type of Pharmacy
दुकान का प्रकार

- Independent स्वतंत्र ☐
- Attached to any health facility : किसी संस्थान से संलग्न ☐
- Whole sale थोक विक्रेता ☐
- Retail खुदरा विक्रेता ☐

Main system of medicine dispensed :
आप मुख्य रूप से किस चिकित्सा पद्धति की दवाई वितरित करते हैं

- Allopathy एलोपैथी (अंग्रेजी दवाई) ☐
- Ayurveda आयुर्वेद ☐
- Homeopathy होम्योपैथी ☐
- Unnani यूनानी ☐
- Siddha सिद्धा ☐
- or any other (specify) या अन्य (विवरण दें) ☐

Apart from this are you dispensing medicine of any other system of medicine ? Yes/No.
क्या आप इसके अलावा भी अन्य किसी चिकित्सा पद्धति की दवाई वितरित करते हैं ? हां /नहीं

If Yes (✓appropriate)
यदि हां (✓ का चिन्ह लगाईये)

- Allopathy एलोपैथी (अंग्रेजी दवाई) ☐
- Ayurveda आयुर्वेद ☐
- Homeopathy होम्योपैथी ☐
- Unnani यूनानी ☐
- Siddha सिद्धा ☐
- or any other (specify) या अन्य (विवरण दें) ☐

Do you dispense vaccines or immunologicals in this pharmacy ? Yes/No.
क्या आप वैक्सीन/ रोग प्रतिबंधक दवाईयाँ भी वितरित करते हैं ? हां /नहीं

If Yes (✓appropriate)
यदि हां (✓ का चिन्ह लगाईये)

- OPV / पोलियो ☐
- BCG / बीसीजी ☐
- DPT / डीपीटी ☐
- DT / डीटी ☐
- TT / टीटेनस ☐
- Measles / खसरा ☐
- Hepatitis A / पीलिया A ☐
- Hepatitis B / पीलिया B ☐
- Vitamin A / विटामीन ए ☐
- Rabies Vaccines/ रेबिज टीका ☐
- Antirabies serum / रेबिज प्रतिजैविक ☐
- Antisnake Venom /सांप कांटे की दवा ☐
- Pneumococcal vaccine ☐
- Yellow fever ☐

Other vaccines / अन्य टीके ....

Details of the staff :
कर्मचारियों का जानकारी –

Name नाम	Age आयु	Desig. पद	Qualification शैक्षणिक योग्यता	M/F पु / म

Whether veterinary medicines are also available in this pharmacy ? Yes/ No.
क्या पशुचिकित्सा की दवाएँ भी इस दुकान में उपलब्ध है ? हाँ /नहीं

Any distinguishing feature of this pharmacy
आपकी दवा दुकान की खास विशेषता

Survey by : Name Signature

Date

Checked by
