# Supplementary material for: Mapping private pharmacies and their characteristics in Ujjain district, Central India
Source: BMC Health Serv Res. 2011 Dec 28;11:351. doi: 10.1186/1472-6963-11-351 (PMC3272060; doi:10.1186/1472-6963-11-351)
Supplement: Additional file 2 — Questionnaire 2. Field questionnaire for tracer medicines available in pharmacy. [file 1472-6963-11-351-S2.PDF]

R.D.Gardi Medical College, Ujjain.
Proforma: Medicines available in the pharmacies

Name and address:

Tick (√) the drugs available in this pharmacy.

Note: Few common brand names are suggested as a clue but you can Tick (√) even if any brand other than the indicated ones are available

Formulation	Medicine	Common Brand Names	Tick (√) the drugs available
Oral (Tab / Cap / Syrup / Drops)	Trimethoprime-Sulphamethoxazole	SEPTRAN	
	Ampicillin / Amoxycillin	MOX / AMOX	
	Ampicillin + Clavulanic Acid	AUGMENTIN / FLEMICLAV / CLAVAM / MOXIKIND	
	Ciprofloxacin	CIPLOX / CIFRAN – OD / ZOXAN / CIPROBID	
	Anti TB (Tuberculosis) (Isoniazid / Rifampicin)	AKURIT / AKT-4 / MECOX + / FORCOX /	
	Prednisolone	WYSOLONE / OMNACODRIL	
Injections	Cefotaxime	Inj TAXIM / C-TAX / OMNATAX / NOVATAX	
	Hydrocortisone	PRIMACORT / EFFCORLIN	
	Dexamethasone	DEXA / DEXONA	
IV Fluids	Saline, glucose, dextrose, ringer lactate	NS, DNS, RL, D5	
